# Cell cycle regulator MYBL2 is a distinct vulnerability in acute myeloid leukemia

**DOI:** 10.1038/s41420-025-02810-4

**Published:** 2025-10-20

**Authors:** Sandra Küchler, Silke Brilloff, Silvia Schäfer, Elahe Rahimian, Vida Kufrin, Shraddha S. Peri, Julian Musa, Thomas G. P. Grünewald, Denis M. Schewe, Claudia R. Ball, Martin Bornhäuser, Hanno Glimm, Marius Bill, Alexander A. Wurm

**Affiliations:** 1 Mildred Scheel Early Career Center, National Center for Tumor Diseases (NCT/UCC) Dresden, Faculty of Medicine and University Hospital Carl Gustav Carus, TU Dresden University of Technology, Dresden, Germany; 2 Department for Translational Medical Oncology, National Center for Tumor Diseases Dresden (NCT/UCC), a partnership between DKFZ, Faculty of Medicine and University Hospital Carl Gustav Carus, TU Dresden University of Technology, and Helmholtz-Zentrum Dresden - Rossendorf (HZDR), Dresden, Germany; 3 Department of Pediatric Hematology and Oncology, University Hospital Dresden and National Center for Tumor Diseases (NCT/UCC) Dresden, Dresden, Germany; 4 Department of Internal Medicine I, University Hospital Carl Gustav Carus, TU Dresden University of Technology, Dresden, Germany; 5 Department of General, Visceral, Thoracic, and Transplant Surgery, University Hospital of Giessen and Marburg, Giessen, Germany; 6 Division of Translational Pediatric Sarcoma Research, German Cancer Research Center (DKFZ), German Cancer Consortium (DKTK), Heidelberg, Germany; 7 Hopp Children’s Cancer Center (KiTZ), Heidelberg, Germany; 8 National Center for Tumor Diseases (NCT), NCT Heidelberg, a partnership between DKFZ and Heidelberg University Hospital, Heidelberg, Germany; 9 Institute of Pathology, Heidelberg University Hospital, Heidelberg, Germany; 10 Translational Medical Oncology, Faculty of Medicine and University Hospital Carl Gustav Carus, TU Dresden University of Technology, Dresden, Germany; 11 German Cancer Consortium (DKTK), partner site Dresden, Dresden, Germany; 12 TU Dresden University of Technology, Faculty of Biology, Dresden, Germany; 13 Translational Functional Cancer Genomics, German Cancer Research Center (DKFZ), Heidelberg, Germany

**Keywords:** Acute myeloid leukaemia, Senescence

## Abstract

Acute myeloid leukemia (AML) is a hematologic malignancy characterized by the accumulation of myeloid blasts in the bone marrow. Despite the availability of potential curative treatments, patients frequently experience unfavorable outcomes. One crucial aspect contributing to relapse is the plasticity of leukemic clones, which enables them to switch between active proliferation and dormancy. The adaptability of AML underscores the need for novel therapies targeting AML-specific proteins. To address this, genome-wide CRISPR screens can be utilized to identify cancer entity-specific vulnerabilities. Leveraging publicly available functional genomics datasets and comparing AML with non-AML cancer cell lines, we identified a significant dependency on the cell cycle-regulating gene *MYBL2* in AML. We describe MYBL2 as a key driver of AML cell growth and proliferation, highlighting its established role as a cell cycle regulator. Also, our findings uncover its previously unrecognized function as an inhibitor of cellular senescence. A knockdown of *MYBL2* induces cell cycle arrest in the G2/M phase with subsequent induction of apoptosis in vitro, and reduces leukemic burden in a patient-derived xenograft (PDX) model in vivo. Interestingly, some AML cells evade apoptosis and enter a senescent-like phenotype upon *MYBL2*-knockdown, which is reversible upon re-expression of *MYBL2*. Finally, analyses of clinical data from two publicly available patient cohorts demonstrate a lower probability of survival in patients with higher *MYBL2* expression, further hinting at the potential relevance of MYBL2 in AML. In conclusion, our findings demonstrate the essential role of MYBL2 in AML, governing the balance between cell proliferation, cell survival and senescence, ultimately influencing the fate of AML cells.

## Introduction

Acute myeloid leukemia (AML) is a heterogeneous malignancy of the hematopoietic system characterized by a differentiation block and an abnormal accumulation of malignant progenitors in the bone marrow and, to some extent, other organs [1]. The standard of care therapy for fit AML patients has not substantially changed in the last decades and consists of an induction chemotherapy (cytarabine plus an anthracycline) followed by a consolidation therapy or hematopoietic stem cell transplantation. Despite high initial response rates, a significant number of patients experience relapse, and the prognosis for AML remains poor. Moreover, the standard therapy for AML causes significant toxic side effects and is often not recommended for older patients [1–3]. In recent years, increasingly individualized alternative treatment options have emerged, initiating the era of precision oncology. These targeted therapies aim to combine a lower toxicity of the treatment with an effective anti-leukemia effect. Some of them, such as the BH3 mimetic venetoclax, tyrosine kinase inhibitors, or hypomethylating agents, have been approved and have increased overall survival in some patient groups. However, relapse rates and therapy resistance remain high [4–9]. Therefore, identifying additional AML targets is crucial for developing novel therapies. To address this, genome-wide CRISPR screens can facilitate the discovery of cancer entity-specific vulnerabilities [10–12]. Resources such as the Dependency Map (DepMap) provide data from tumor cell lines across various cancer types, derived from genome-wide RNAi and CRISPR loss-of-function screens to identify genes essential for cell growth [13].

In the present study, we identified MYBL2 as a key cell cycle regulator and essential for the proliferation of AML cells in vitro and in vivo. A knockdown of *MYBL2* induces apoptosis and a senescence-like phenotype starting from an arrest at the G2/M phase of the cell cycle. The observed phenotype was reversible upon re-expression of *MYBL2*. Publicly available patient data show a decreased survival with high *MYBL2* expression and an expression of senescence-inhibiting genes within that patient group, underlining the relevance of our findings in a clinical setting. Our investigations suggest that MYBL2 is an essential transcription factor for AML cells, which determines the fate between active and senescent AML cells.

## Results

### *MYBL2* is a vulnerability gene candidate in AML

**Fig. 1:**
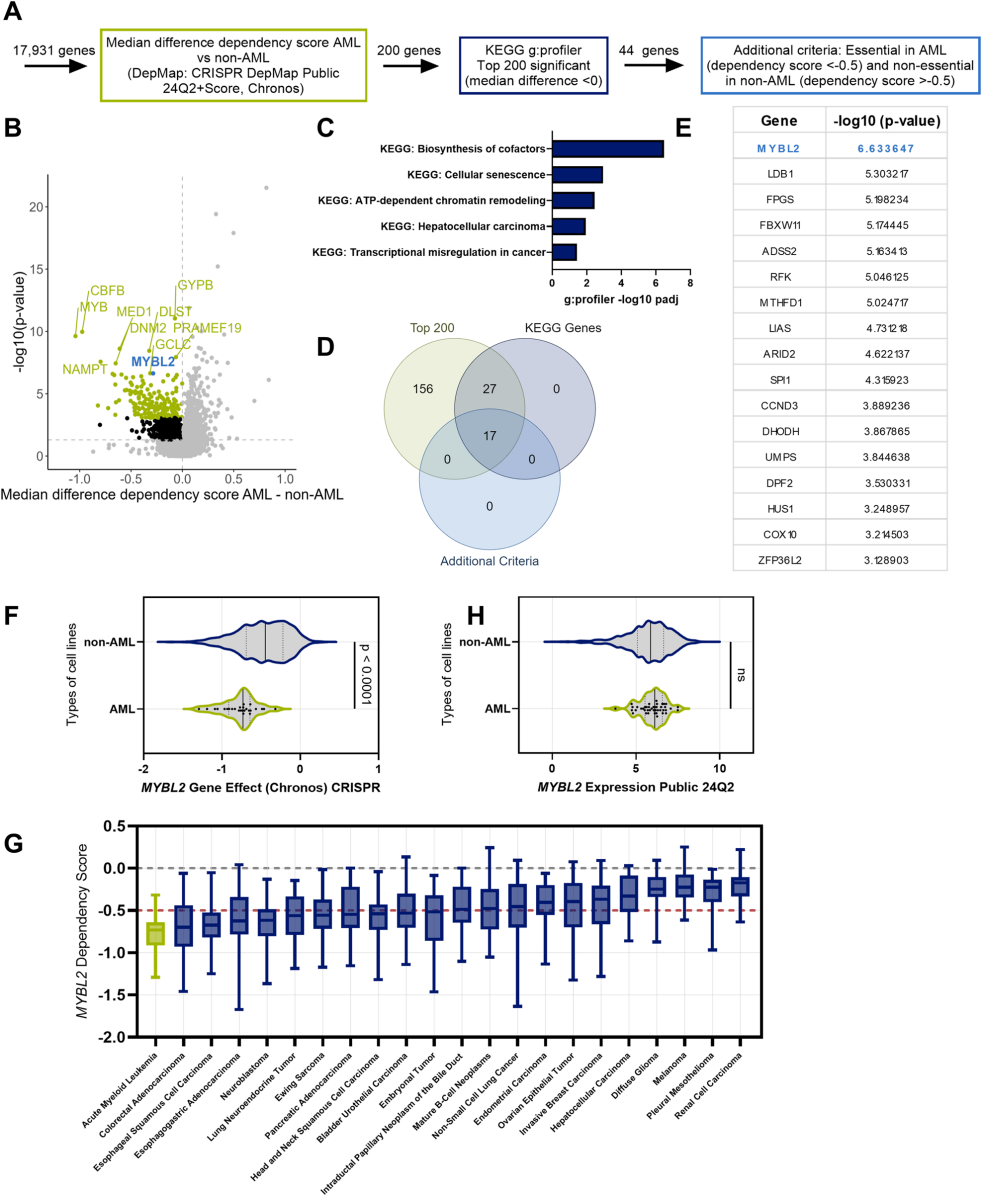
Comprehensive analysis using the DepMap essentiality screen dataset identifies *MYBL2* as a potential target in AML. **A** Workflow of potential target selection from the CRISPR-Cas9 24Q2 (Chronos) dataset (DepMap). **B** Analysis of the DepMap’s public genome-scale CRISPR-Cas9 24Q2 (Chronos) essentiality screen dataset comparing dependency scores of AML cell lines (*n* = 26) to cancer cell lines of other entities (*n* = 1124). Volcano plot represents the median difference between AML cell lines and non-AML cell lines for each gene (*n* = 17,931) and the significance (−log10 *p* value). In green are the top 200 most significant genes with a median difference below 0. Analysis and *t* test were performed with R version 4.3.2. **C** KEGG terms from pathway enrichment analysis using g:Profiler obtained from the top 200 ranked genes. **D** Overlaps from each step in the analysis are represented in a Venn diagram. **E** Genes from (**C**) filtered for genes essential in AML (dependency score <−0.5) and non-essential in non-AML (dependency score >−0.5), ranked based on significance. **F** Violin plot of *MYBL2* dependency scores from the DepMap data set. In green are all AML cell lines (*n* = 26). In blue are all other included cancer cell lines (*n* = 1076). The line shows the median for each, and the quartiles are represented as dotted lines, two-sided *t* test. **G** Box plot representing dependency scores across cancer entities. Cancer entities that had a minimum of 15 cell lines within the data set were included and ranked based on the median dependency score. Data from all cell lines can be found in supplementary Table 2. AML is highlighted in green. **H** Violin plot of *MYBL2* expression from the DepMap Expression_Public_24Q2 data set in AML cell lines (*n* = 43) (green) and in non-AML cell lines (*n* = 1394) (blue).

To identify potential gene candidates with a specifically high dependency in AML, we conducted a comprehensive analysis using data from the “Cancer Dependency Map” project. In this dataset, the primary metric is the dependency score, which reflects the relative impact of target gene perturbation retrieved by large-scale functional CRISPR screening approaches on cell proliferation, normalized for each cell line. Lower scores indicate greater dependency, with scores below −0.5 for individual genes typically considered indicative of complete essentiality [12, 13]. To identify potential specific vulnerabilities in AML, we designed a distinct target selection workflow based on the following criteria (Fig. 1A). First, we compared the median dependency scores of all included AML cell lines (*n* = 26) with the median scores of all other cancer cell lines (*n* = 1124) for in total 17,931 genes. From these calculated differences, we selected the top 200 significant genes with a stronger dependency in AML compared to other cancers. These genes were then subjected to functional enrichment analysis using KEGG pathways [14, 15] by g:Profiler [16]. Next, the identified genes within these pathways were considered for further refinement to focus on AML-specific vulnerabilities. Genes with median dependency scores <−0.5 in AML cell lines were retained, while those with scores >−0.5 in non-AML cell lines were kept to exclude genes essential to non-AML cell lines (Supplementary Table 1). From the 200 most significant genes, 44 genes were part of the KEGG pathways (Fig. 1B). This approach yielded only five KEGG pathways enriched in AML cell lines compared to non-AML cancer cell lines (Fig. 1C). Of note, cellular senescence appeared to be a top signature distinct to AML. Within those five KEGG annotations, 17 AML-specific vulnerability genes remained after they were subjected to our selection criteria, with MYBL2 as the most significant hit (Fig. 1D, E). MYBL2 (MYB proto-oncogene-like 2) is a cell cycle regulator and is involved in the proliferation of healthy hematopoietic stem cells; thus, it might be a proto-oncogene that can develop into an oncogene by deregulation, driving cancer initiation and progression [17, 18]. Numerous prior studies documented that MYBL2 is involved in driving cancer cell growth in colorectal cancer [19], in gallbladder cancer [20], adenocarcinoma [21], and ovarian cancer [22]. In AML, *MYBL2* expression has been described as a negative prognostic factor [23], but its role has not been further explored. Consequently, we sought to investigate whether MYBL2 plays a similar role in driving cancer growth in the context of AML. When comparing *MYBL2* dependency scores in AML with all other included cancer cell lines within the dataset, AML cell lines exhibit significantly higher dependency (Fig. 1F, G, Supplementary Table 2). Next, we investigated whether the dependency could be attributed to *MYBL2* expression levels. However, no significant difference could be observed between AML and non-AML cell lines (Fig. 1H).

Taken together, our findings establish MYBL2 as a compelling candidate for further investigation.

### AML cells are dependent on *MYBL2* expression

**Fig. 2:**
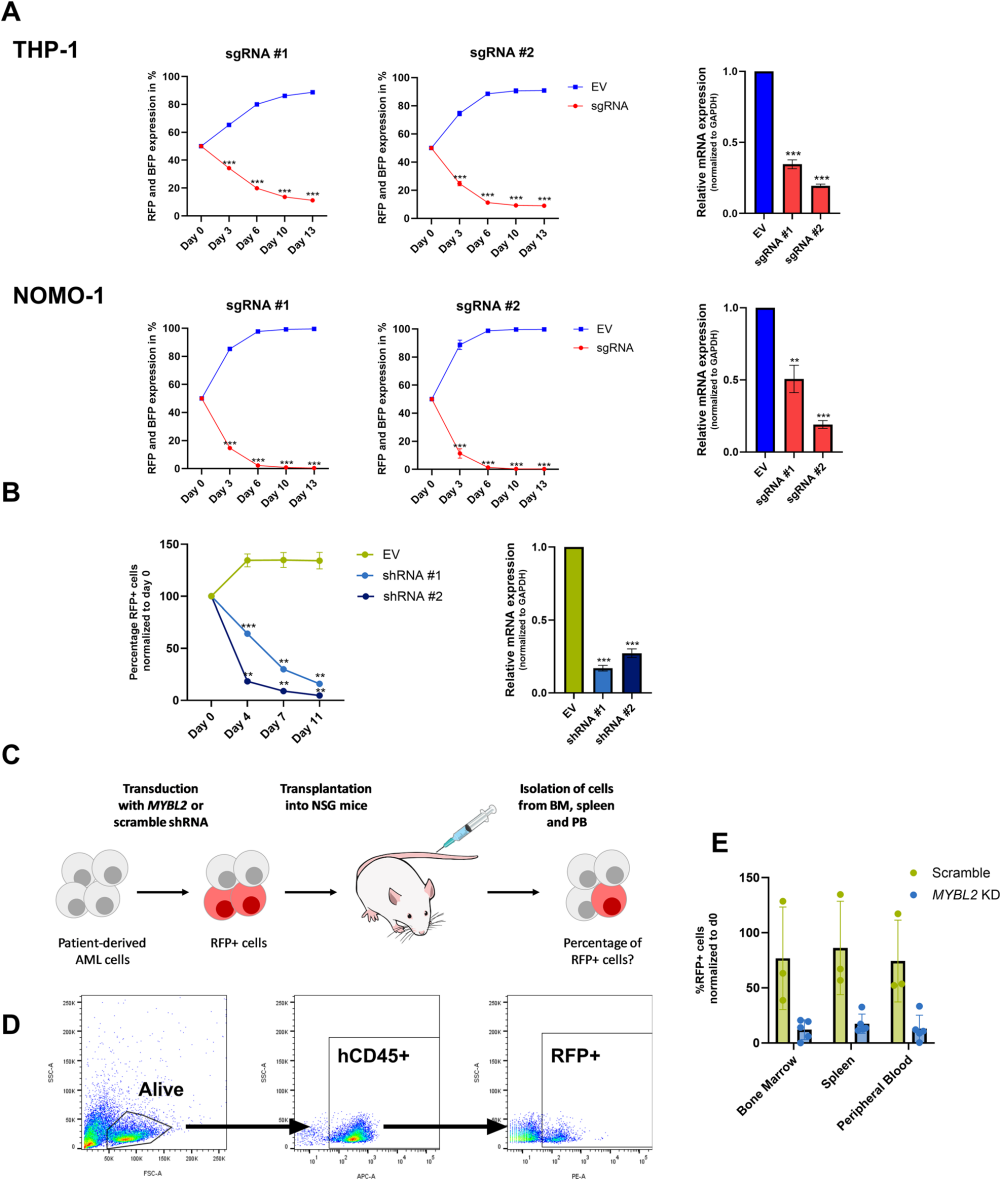
AML cells are dependent on *MYBL2* expression. **A** CRISPRi growth competition assay of sgRNA (targeting *MYBL2*) transduced THP-1 and NOMO-1 cells against empty vector (EV) transduced cells. Flow cytometry data were measured at four different time points, showing the percentage of RFP and BFP expression for sgRNA transduced cell populations and EV transduced populations, respectively. *MYBL2* mRNA expression levels following knockdown of *MYBL2* as measured by qPCR. Expression relative to EV and normalized to GAPDH. Indicated *p*-values were determined by a two-tailed t-test and summarized three independent biological replicates **p < 0.01, ***p < 0.001. **B** Growth competition assay of shRNA or EV transduced THP-1 cells against the untransduced population. Indicated *p* value was determined by a two-tailed *t* test and summarized three independent biological replicates **p < 0.01, ***p < 0.001. *MYBL2* mRNA expression levels following knockdown of *MYBL2* as measured by qPCR. Expression relative to EV and normalized to GAPDH. Indicated *p* values were determined by a two-tailed t-test and summarized three independent biological replicates ***p < 0.001. **C** Graphical workflow of the in vivo competition assay using AML PDX transduced with shRNA scramble and shRNA *MYBL2,* respectively. **D** Gating strategy- RFP+ cells were gated from the hCD45+ population. **E** Flow cytometry analysis for RFP+ cells gated from hCD45+ cells from bone marrow, spleen and peripheral blood, normalized to initial transduction efficiency on day 0. *N* = 3 for shRNA scramble and *n* = 5 for shRNA *MYBL2*.

To assess the functional role of MYBL2 in AML, we conducted CRISPR interference (CRISPRi) knockdown experiments in the two commonly used AML models THP-1 and NOMO-1. According to DepMap, these models exhibit a high (THP-1: −0.85) and intermediate (NOMO-1: −0.56) *MYBL2* dependency. We performed flow cytometry-based growth competition assays to investigate the effect of a *MYBL2* knockdown. Here, dCas9-KRAB fusion protein-expressing THP-1 or NOMO-1 cells were transduced with sgRNAs targeting *MYBL2*, each labeled with RFP, or an empty vector (EV) control labeled with BFP. Cells were initially seeded at a 1:1 ratio, and their growth was monitored over a period of two weeks. Across the cell lines, the knockdown of *MYBL2* resulted in decreased cell growth, and in contrast, the BFP+ control cells overgrew the RFP-labelled *MYBL2* knockdown counterparts. The knockdown efficiency was confirmed by qPCR (Fig. 2A). Similar results were observed for OCI-AML2, a cell line with a comparable DepMap dependency score to NOMO-1 (Supplementary Fig. 1A). We confirmed these results by using shRNAs targeting *MYBL2* in THP-1 (Fig. 2B). Given the functional role of *MYBL2* in cell lines in vitro, we sought to validate the effect of *MYBL2* deficiency in a more clinically relevant model using AML PDX cells in vivo. Here, PDX cells from an AML patient were transduced with a *MYBL2*-targeting shRNA or a scramble shRNA control to achieve a transduction efficiency of ~50%. Subsequently, the PDX cells were transplanted into immunodeficient NOD-Scid-Gamma (NSG) mice to conduct an in vivo competition assay. The engraftment of the cells was monitored via blood collections and measuring hCD45+ cells by flow cytometry. After successful engraftment and leukemia onset, mice were sacrificed, and cells from bone marrow, peripheral blood, and spleen were extracted and used for flow cytometry analysis to determine the distribution of RFP+ cells within the fraction of human hCD45+ AML cells (Fig. 2C, D). We observed a reduced amount of *MYBL2* knockdown cells contributing to AML onset in all tissues, confirming results obtained in vitro (Fig. 2E, Supplementary Fig. 1B). Taken together, our results show that *MYBL2* is essential for AML cell growth in vitro and in vivo.

### *MYBL2* knockdown induces a G2/M cell cycle arrest, apoptosis and senescence in AML

**Fig. 3:**
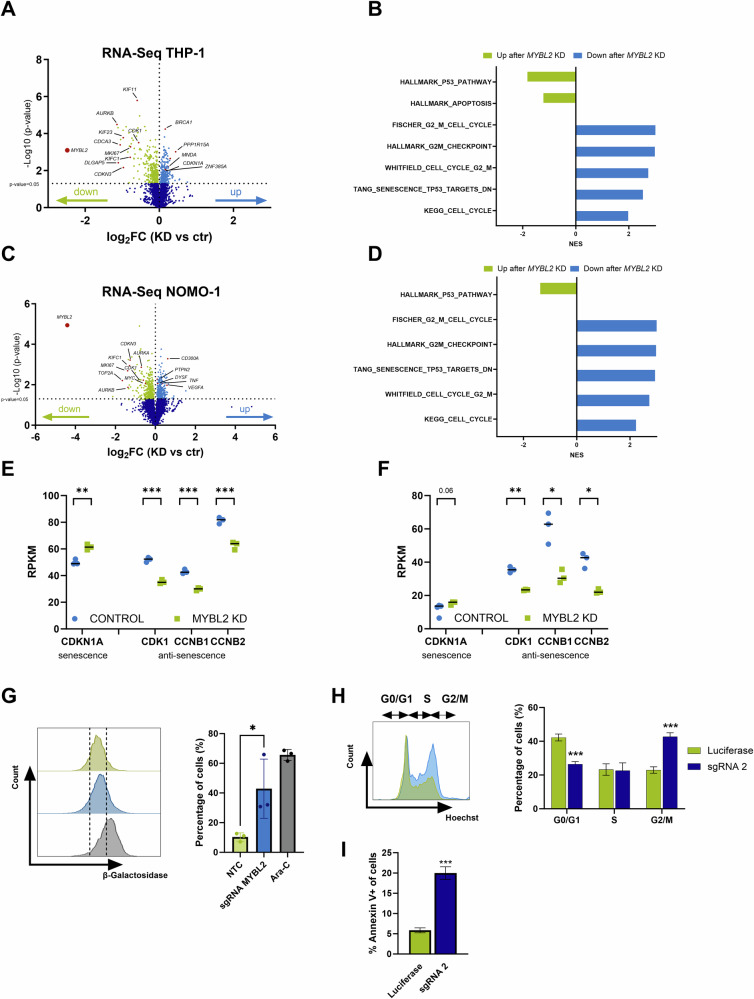
MYBL2 regulates cell cycle and senescence in AML cell lines. **A** Transcriptomics by RNA sequencing is shown as a volcano plot representing differential gene expression between sgRNA luciferase (control) and sgRNA #2-MYBL2 (knockdown) transduced THP-1 cells and **C** NOMO-1 cells five days after lentiviral transduction. Genes with significantly decreased expression are shown in green. Genes with significantly increased expression are depicted in light blue (*p* < 0.05). *N* = 3. **B** Gene set enrichment analysis (GSEA) depicting normalized enrichment scores (NES) for THP-1 and **D** NOMO-1. **E** Expression levels of senescence driver gene *CDKN1A* and senescence-inhibiting genes *CDK1*, *CCNB1*, and *CCNB2* in THP-1 and **F** NOMO-1 cells from RNA sequencing. **G** Senescence analysis in THP-1 by measurement of active β-galactosidase. Fluorescence intensity was determined by flow cytometry for non-targeting control (NTC), sgRNA MYBL2 and treatment with cytarabine (Ara-C) as a positive control. Gates were set for each replicate individually based on the NTC and divided into high and low signal. *P* value was determined by a two-tailed t-test and summarized three independent biological replicates. *p < 0.05. **H** Gating strategy and cumulative results of cell cycle analysis performed on day 7 after transduction. Color codes correspond to luciferase sgRNA (green) and sgRNA #2 (dark blue). *P* value was determined by a two-tailed *t* test and summarized three independent biological replicates. ***p < 0.001. **I** Apoptosis analysis by flow cytometry measurement of Annexin V 7 days after lentiviral transduction of THP-1 with sgRNA luciferase and sgRNA #2. The gating strategy can be found in Supplementary Fig. 2B. *P* value was determined by a two-tailed t-test and summarized three independent biological replicates. *p < 0.05, **p < 0.01.

The previous results demonstrated that AML cell growth depends on *MYBL2*. However, the exact mechanisms remained unclear. To determine the downstream effectors regulated by MYBL2, transcriptome analysis by RNA sequencing was performed in THP-1 and NOMO-1 after CRISPRi-mediated knockdown of *MYBL2* (Supplementary Table 3). Here, we identified 650 differentially expressed genes (DEGs, *p* < 0.05), with 337 downregulated and 313 upregulated in THP-1 cells (Fig. 3A). For NOMO-1, a total of 642 DEGs were identified, among which 359 were downregulated and 283 were upregulated (Fig. 3C). As anticipated, the strongest effect was observed for *MYBL2* itself in both models confirming successful gene silencing. *MYBL2* knockdown led to a significant downregulation of cell cycle genes such as *AURKB*, *CDKN3*, *KIF11*, *KIFC1*, and *MKI67*. To determine whether other MYB gene family members [24] are regulated by *MYBL2*, we evaluated the expression of *MYB* and *MYBL1* and found no significant changes, indicating that *MYBL2* does not regulate these genes in our AML models. To identify altered gene signatures and expression profiles, we performed Gene Set Enrichment Analysis (GSEA). Both models revealed that downregulated DEGs were involved in cell cycle and mitotic processes. Additionally, a correlation with the gene signature for HALLMARK_P53_pathway has been identified. Furthermore, we observed a significant enrichment for TANG-Senescence-TP53-targets-DN in the control cells. In THP-1, apoptosis was also identified as an upregulated signature upon *MYBL2* knockdown (Fig. 3B, D, Supplementary Fig. 2A). Since we observed senescence signatures as major hallmarks specific to AML, we were interested in the expression of senescence-associated genes upon *MYBL2* repression. We observed an upregulation of the senescence driver *CDKN1A* (*p21*), and a downregulation of senescence-inhibiting genes *CDK1*, *CCNB1*, and *CCNB2* in both cell models (Fig. 3E, F).

As RNA sequencing data suggest that *MYBL2* acts as a negative regulator of senescence, we quantified the frequency of senescent cells upon *MYBL2* depletion using an active β-galactosidase flow cytometry assay in THP-1 cells. As a positive control, cells were treated with 1 µM cytarabine (Ara-C). It has been previously reported that treatment of AML cells with cytarabine induces a senescence phenotype [25]. We found an increase of β-galactosidase activity upon knockdown of *MYBL2*, indicating the induction of a senescence-like phenotype upon *MYBL2* inhibition (Fig. 3G).

Since our transcriptomic data showed evidence for changes in cell cycle and survival upon *MYBL2* knockdown, we also conducted cell cycle and apoptosis analysis. Exemplarily, these experiments were performed in THP-1 cells. Here, we observed a significant decrease in the proportion of cells in G0/G1 and a concomitant increase in G2/M upon knockdown (Fig. 3H). Thus, the reduced ability of *MYBL2*-knockdown cells to proliferate can be explained by a partial arrest in G2/M. In agreement with this finding, the knockdown of *MYBL2* also led to a significant increase in the number of apoptotic cells (Fig. 3I). Collectively, our findings demonstrate that MYBL2 regulates cell cycle, cell survival and senescence in AML.

### Effects of *MYBL2* downregulation are reversible

**Fig. 4:**
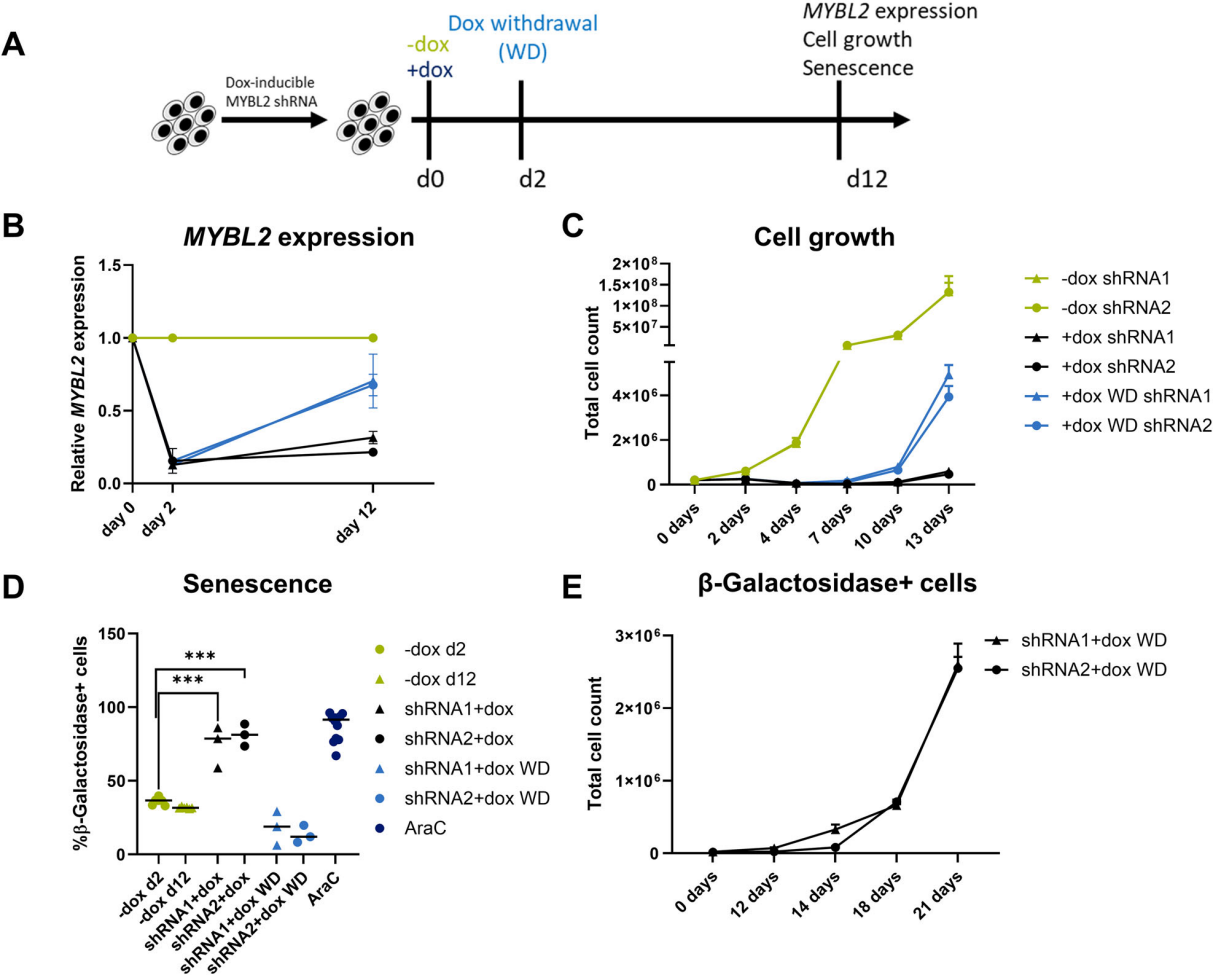
*MYBL2* knockdown-related effects are reversible. **A** Experimental layout of knockdown experiments with TET-inducible shRNAs targeting *MYBL2* (shRNA #1 and #2). *MYBL2* knockdown was induced on day 0 by adding doxycycline (+dox). The control condition (−dox) was seeded at the same time. Dox was withdrawn (WD) on day 2, and cells were kept in culture until day 12. *MYBL2* expression, cell growth and senescence were monitored. **B** Timeline of relative *MYBL2* expression measured by qPCR. Expression relative to –dox control and normalized to GAPDH. **C** Growth curve of cells obtained by cell counting. Conditions: without doxycycline (-dox), with continuous doxycycline (+dox) and withdrawal of doxycycline (WD) after 48 hours. The experiment was performed in independent triplicate for each shRNA and condition. **D** Measurement of β-galactosidase by flow cytometry. Fluorescence intensity was determined for both shRNAs by flow cytometry for −dox, +dox, WD and treatment with Ara-C as a positive control. *P* value was determined by a two-tailed t-test and summarized three independent biological replicates. ***p < 0.001. **E** Dox-induced cells with the highest signal for active β-galactosidase were sorted in triplicate, reseeded and counted at different time points. Results are represented as a growth curve.

Given the recent observation that senescence can be reversible in AML [25], we sought to determine whether AML cells could recover and regrow once the knockdown was reversed. To address this, we utilized a TET-inducible shRNA system [26] in THP-1 cells. Here, *MYBL2*-knockdown can be induced by adding doxycycline (dox) to the medium and reversed by removing dox. *MYBL2* knockdown was induced for 48 h ( + dox) followed by dox withdrawal (WD) and further culture for 12 days (Fig. 4A). We assessed *MYBL2* expression, cell growth and cellular senescence after dox induction and WD. *MYBL2* expression decreased upon dox induction and increased again after WD (Fig. 4B). Consistently, cell growth was reduced during knockdown, but resumed after WD (Fig. 4C). β-Galactosidase analysis was conducted to determine if a re-expression of *MYBL2* would reverse the senescence phenotype caused by the knockdown. Senescence levels increased two days after induction of the knockdown, but decreased after WD, even to lower levels compared to the control condition (Fig. 4D and Supplementary Fig. 3). To prove that the regrowth is really mediated by the senescent population, we sorted β-Galactosidase+ cells two days after the induced knockdown of *MYBL2*, and cultured them in the absence of dox for up to three weeks. After 12 days, cells started to regrow (Fig. 4E). In summary, our findings demonstrate the reversibility of *MYBL2* knockdown effects, specifically the reduced growth as well as the senescence-like phenotype.

### High expression of *MYBL2* in AML patients is associated with poor overall survival and linked to cell cycle progression and inhibition of senescence

**Fig. 5:**
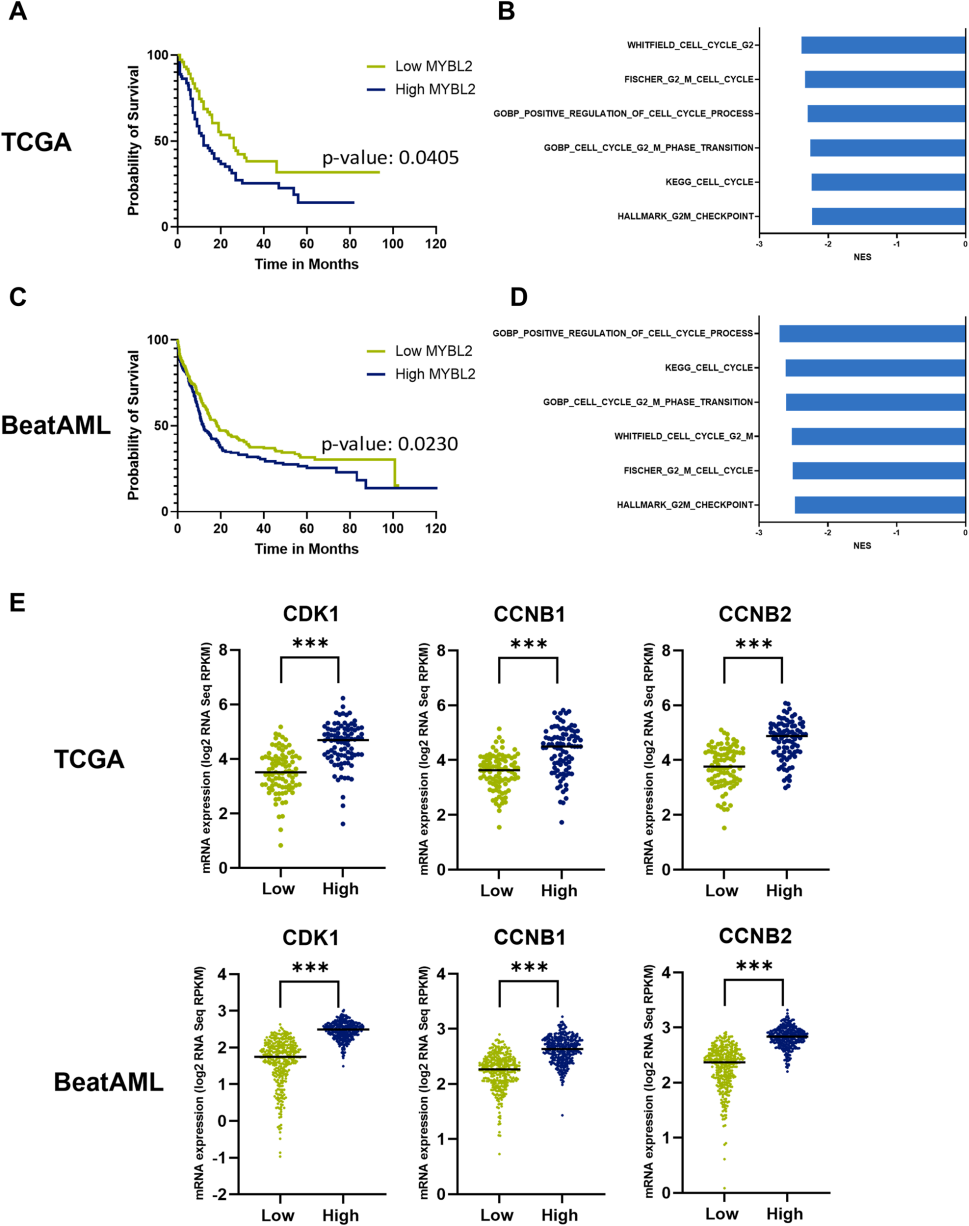
High *MYBL2* expression is linked to worse overall survival, cell cycle processes and high expression of senescence-inhibiting genes. **A** Kaplan–Meier survival analysis based on the median (low and high) *MYBL2* gene transcript expression in AML with patient data from TCGA (179 patient samples) and **C** Beat AML (671 patient samples). Significance was assessed with the Log-Rank test. **B** Gene set enrichment analysis (GSEA) indicating enrichment for gene signatures related to cell cycle in high *MYBL2* expressing patients within the TCGA cohort and **D** Beat AML cohort. **E** Expression levels of CDK1, CCNB1, CCNB2 in AML patients from TCGA and Beat AML were stratified according to their median *MYBL2* expression into two groups (low versus high). ***p < 0.001 two-tailed t test.

Lastly, we aimed to investigate the association between *MYBL2* expression and the overall survival of AML patients by examining publicly available AML patient data from The Cancer Genome Atlas (TCGA) [27] and the Beat AML cohort [28]. We categorized the patients into two groups: a high *MYBL2* expression group and a low *MYBL2* expression group, using the median *MYBL2* expression as a cutoff. In both cohorts, we observed a significantly lower overall survival in patients with high *MYBL2* levels (Fig. 5A, C).

In addition, we were interested in whether high- and low-expressing patient groups also show differences in gene expression signatures related to the cell cycle. Indeed, GSEA revealed higher expression of gene signatures related to cell cycle in the *MYBL2* high groups in both cohorts, suggesting that a high *MYBL2* expression is causing a more aggressive disease phenotype (Fig. 5B, D).

Moreover, we aimed to investigate the potential correlation between elevated *MYBL2* expression and the expression of senescence-inhibiting genes *CDK1*, *CCNB1*, and *CCNB2* in these AML cohorts, which we identified as being downregulated after *MYBL2* knockdown in our transcriptome data. As expected, all three of these specified genes exhibited significantly higher expression levels in patients with elevated *MYBL2* expression in both study cohorts (Fig. 5E). Altogether, we identified a correlation between high *MYBL2* expression and a worse overall survival in AML patients, as well as its association with cell cycle progression and the expression of anti-senescence genes, underscoring the clinical significance of *MYBL2* expression.

## Discussion

In this study, we aimed to identify a new target gene to expand the spectrum of AML-specific vulnerabilities and biomarkers for potential treatment options. We introduce MYBL2, a transcription factor involved in the development of various tumor types and correlated with reduced survival rates, as a potential novel specific vulnerability in AML. The function of MYBL2 in normal cell physiology, such as regulation of cell cycle, proliferation and cell survival, suggests that MYBL2 could be a proto-oncogene which can develop into an oncogene by deregulation, driving cancer initiation and progression [17, 29, 30]. Numerous prior studies documented that MYBL2 is involved in driving cancer cell growth [20, 22, 31]. In the context of AML, Fuster and colleagues have described an increased expression of MYBL2 compared to normal hematopoietic stem cells and defined a negative prognostic role of MYBL2 overexpression [23]. Wang et al. identified MYBL2 as a downstream regulator of S1PR1 in AML, highlighting its potential role in leukemic cell signaling and mediating the stemness of leukemia stem cells [32]. Additionally, Sun and colleagues demonstrated that circular MYBL2 RNA derived from MYBL2 was associated with an increased rate of proliferation in FLT3-mutated AML [33]. Consequently, we sought to further investigate the functional role of MYBL2 in AML in relevant pre-clinical models.

Within our study, we identified dependency of AML cell lines and a PDX model on *MYBL2* expression, which was in line with findings utilizing data from the CRISPR DepMap dataset [12, 13]. Cell cycle progression and the evasion of apoptosis are key features in cancer development [34]. By RNA sequencing, we observed that cell cycle-regulating genes were downregulated upon knockdown of *MYBL2*. In concordance, a block of *MYBL2* caused an accumulation of cells in G2/M. These findings therefore indicate a role of MYBL2 as a mediator of late cell cycle genes. The results are supported by other studies demonstrating that MYBL2 regulates the expression of several key genes required for G2/M transition [35, 36]. It has been reported that a G2/M arrest was the result of a *MYBL2* knockdown in human glioma [37], in melanoma [38], in mouse embryonic stem cells [39] and in human fibroblasts [40]. Our experiments revealed an increase in apoptotic cells upon *MYBL2* knockdown, confirming its role in cell survival. Consistent results showing an increase in apoptosis were obtained in studies knocking down *MYBL2* in other cancer types, such as non-small-cell lung cancer [31] and hepatocellular carcinoma [41].

Given our findings from the KEGG pathway analysis as well as prior studies on MYBL2 and its role in senescence [42], we investigated this relationship in AML. Previous research showed that MYBL2 gets downregulated upon senescence and that MYBL2 represses senescence in fibroblasts [43–45] and cervical cancer cells [45]. In our findings, we observed an increase in senescent cells upon *MYBL2* knockdown. Utilizing GSEA, we found associations with the p53 pathway, which is also known to regulate senescence [46, 47]. *TP53*-mutated cancers are associated with MYBL2 activation and deregulation of cell cycle progression and cell survival [48–50].

Senescence in hematopoietic stem cells is closely linked to aging and involves DNA damage, telomere shortening, and oxidative stress, leading to reduced regenerative capacity, which contributes to increased disease susceptibility and risk of leukemias [51–53]. In AML, senescence can be induced within the bone marrow microenvironment, which can promote leukemic cell survival and lead to disease progression [54]. Senescence has been described as a stable growth arrest with a secretion of pro-inflammatory cytokines and chemokines known as the senescence-associated secretory phenotype (SASP) [55–57]. Originally, senescence was considered an irreversible arrest of cell proliferation [58]. This assumption was initially extended to other senescence phenotypes such as oncogene-induced senescence, therapy-induced senescence, or virus-induced senescence [59]. However, several recent studies suggest that senescence does not necessarily hinder cancer progression, especially in the context of oncogene-induced or therapy-induced senescence [59–62]. Research by Duy et al. in AML showed that a chemotherapy-induced senescence-like phenotype can be reversed and contribute to leukemia repopulation, thereby describing a resistance mechanism [25, 63]. Following the hypothesis that *MYBL2* knockdown-induced senescence could be reverted, we utilized a doxycycline-inducible shRNA knockdown and observed that the induced phenotype indeed was reversible upon reactivation of *MYBL2*. Further research should explore the therapeutic implications of this phenomenon. Firstly, cells with low *MYBL2* expression may become invisible to chemotherapy. Secondly, regrown cells could exhibit differences, potentially being more resistant. The senescence-like phenotype observed during chemotherapy may be driven by low levels of *MYBL2* and result in disease recurrence if the state is reversible. Thus, we hypothesize that both high and low expression levels of *MYBL2* might contribute to adverse outcomes in patients.

By utilizing data from both TCGA and Beat-AML cohorts, we uncovered a link between high *MYBL2* expression and a poorer overall survival in AML patients, a link to cell cycle progression, as well as the expression of anti-senescence genes, highlighting the clinical significance of *MYBL2* expression. The findings suggest that AML characterized by elevated *MYBL2* levels is more aggressive, likely due to increased cell proliferation. The prospect of studying MYBL2 and senescence in the future is strengthened by the link between *MYBL2* high-expressing patients and upregulated senescence-inhibiting genes. The data from the patient cohorts imply that *MYBL2* has the potential to serve as a valuable biomarker for predicting patient outcomes and assessing disease aggressiveness. A high *MYBL2* expression has been associated with poor survival in other cancer types, such as hepatocellular carcinoma [64], renal cell carcinoma [65], and breast cancer [66], underlining the clinical relevance of *MYBL2* as a biomarker in several cancer entities. Moreover, it would be interesting to further study how MYBL2 could be of use as a therapeutic target for AML, either through direct inhibition or by targeting potential downstream effectors.

Altogether, our results show that *MYBL2* might be a novel candidate gene for AML-directed therapy. It regulates cell cycle processes, senescence and cell survival in AML. As the phenotype associated with a *MYBL2*-knockdown was reversible, MYBL2 might act as a critical regulator of AML cell plasticity. Future research should focus on characterizing the clinical aspects of this phenomenon, especially focusing on treatment response depending on the level of *MYBL2* expression.

## Materials and methods

### Cancer dependency map (DepMap)

We downloaded CRISPR-Cas9-based dependency data for 1150 pan-cancer cell lines, including 26 AML cell lines, as well as gene expression data for 1437 pan-cancer cell lines, including 43 AML cell lines from the Cancer Dependency Map Project at Broad Institute (https://depmap.org/portal), specifically the CRISPR DepMap Public 24Q2+Score, Chronos dataset and Expression_Public_24Q2 [12, 13]. Raw data were downloaded and analyzed for each cell line. To identify potential vulnerabilities, we compared the median dependency scores of 26 AML cell lines with the median scores of all other cancer cell lines (*n* = 1124) for a total of 17,931 genes. From these calculated differences, the top 200 significant genes with a median difference <0 were selected. Analysis and *p* values (−log10 *p* values) were determined using R version 4.3.2. These genes were subjected to functional enrichment analysis within KEGG pathways [14, 15] using g:Profiler [16]. The identified genes within the pathways were subjected to further criteria. Genes with median dependency scores <−0.5 in AML cell lines were retained, while those with scores >−0.5 in non-AML cell lines were kept to exclude genes essential to non-AML cell lines. Summarized data can be found in Supplementary Tables 1 and 2.

### Cell culture

All cell lines were obtained from the German Collection of Microorganisms and Cell Cultures GmbH (DSMZ). THP-1 cells were cultured in RPMI-1640 medium (ThermoFisher Scientific, Waltham, MA, USA) supplemented with 10% fetal calf serum (FCS, PAN-BIOTEC, Aidenbach, Germany) and 1% penicillin/streptomycin (P/S, Life Technologies, Carlsbad, CA, USA). NOMO-1 cells were maintained in RPMI-1640 with 20% FCS and 1% P/S. OCI-AML2 cells were cultured in Minimum Essential Medium alpha (MEM α; ThermoFisher Scientific) with 10% FCS and 1% P/S. HEK293T cells were cultured in DMEM (ThermoFisher Scientific) supplemented with 10% FCS and 1% P/S. All cells were cultured at 37 °C with 5% CO_2_ in a cell culture incubator. All cell lines were continuously tested for mycoplasma contamination and cross-contamination by Multiplex Cell Authentication (Multiplexion, Heidelberg, Germany).

### Quantitative real-time PCR (qPCR)

RNA extraction, cDNA synthesis and qPCR were performed as described before [67]. Briefly, total RNA was isolated using the RNeasy® Mini Kit (Qiagen, Hilden, Germany) following the manufacturer’s protocol. An optional DNAse digest using the RNase-free DNase Set (Qiagen) was conducted. Reverse transcription for cDNA synthesis was carried out using the RevertAid First Strand cDNA Synthesis Kit (Thermo Scientific). Quantification of mRNA-derived cDNA was performed using the Sso Advanced Universal SYBR Green Super Mastermix (Bio-Rad, Hercules, CA, USA). Quantitative real-time PCR (qPCR) was performed in the C1000 Touch Thermal Cycler (Bio-Rad) in a total volume of 15 µl (7.5 µl Sso Advanced Universal SYBR Green Super Mastermix, 1 µl cDNA, 0.75 µl forward/reverse Primer, 5 µl H_2_O). The sequences of the qPCR primers are listed below. Gene expressions were normalized to *GAPDH* levels, and the relative expression was determined using the 2^(−ΔΔct)^ method.

**Table:** 

Primer	Sequence 5‘–3‘
MYBL2-forward	CACCCACGCTGACGCCTTCGAGCG
MYBL2 reverse	AAACCGCTCGAAGGCGTCAGCGTG
GAPDH-forward	CATCACTGCCACCCAGAAGACTG
GAPDH-reverse	ATGCCAGTGAGCTTCCCGTTCAG

### Production of lentiviral particles and transduction

The transfection was performed using PEIpro® (Polyplus, Illkirch-Graffenstaden, France). 5 × 10^6^ HEK293T cells were seeded in a T75 flask. After 24 h of cultivation, the transfection was performed. A DNA mix containing the 5 µg p103 pMD2.G (addgene), 10 µg p102 pRSV-REV (addgene), 10 µg p101 pMDLg/pRPE (addgene), 10 µg of the plasmid and filled up with DMEM (without FCS) to 250 µl was prepared. The PEIpro mix containing 35 µl PEIpro and 215 µl DMEM (without FCS) was prepared and added to the DNA mix. The mixture was incubated for 15 min and added to the cells. The medium was changed to 13 ml after 4 h. The medium was changed again after 24 h. The lentiviral particles were harvested on days 3 and 4. The supernatant was centrifuged at 2000 × *g* for 15 min and filtered through a 0.45 µm filter. For transduction, the cells were treated with 8 µg/ml polybrene (Millipore, Burlington, MA, USA) before adding the lentivirus.

### CRISPR interference

dCas9-KRAB expressing AML cell lines were generated with lentiviral transduction with pLV hUbC-dCas9 KRAB-T2A-GFP (addgene #67620, a gift from Charles Gersbach). Two sgRNAs targeting *MYBL2* and a non-targeting sgRNA luciferase were cloned into a CRISPRi backbone vector pL.EUP-CRISPRi vector (Eupheria Biotec, Dresden, Germany). Additionally, sgRNA #2 MYBL2 and a non-targeting control (NTC) were cloned into the CRISPRi vector pCRISPRia-v2 (addgene #84832). The sgRNA sequences were designed for targeting *MYBL2* using the CRISPick Broad Institute software. For each gene, two complementary oligonucleotides were synthesized (Biomers.net GmbH, Ulm, Germany). The oligonucleotides were annealed by incubating 1 μl of each oligo (100 μM), 2 μl of 10 × annealing buffer (OriGene Technologies, Inc., Rockville, MD, USA) and 16 μl of ddH2O at 95°C for 5 min, and then cooled for 30 min at room temperature. The pCRISPRia-v2 vector was digested with BIp1 and BstX1 (ThermoFisher Scientific) at 37°C for 1 h, and the pL.EUP-CRISPRi vector with BsmBI at 55°C overnight. The digested vectors were gel-purified using the Zymoclean Gel DNA Recovery Kit (Zymo Research, Irvine, CA, USA). For ligation, 100 ng of the digested vector was combined with 2 ng annealed oligos, 1 μl of 10 × T4 DNA Ligase (Life Technologies), and 2 μl of 5 × ligase reaction buffer (Life Technologies). The ligation mixture was incubated at room temperature for 1 h. The ligated products were transformed into SURE 2 SuperComp Cells *E. coli* (Agilent, Santa Clara, CA, USA) following the manufacturer’s protocol. Individual colonies were picked and grown in LB medium with ampicillin (Sigma-Aldrich, St. Louis, MO, USA). Plasmid DNA was extracted with the ZymoPURE Plasmid Miniprep Kit (Zymo Research). The sgRNA sequences were confirmed with Sanger sequencing (Eurofins Genomics).

pL.EUP-CRISPRi:

sgRNA #1 MYBL2 Top: CACCCACGCTGACGCCTTCGAGCG

Bottom: AAACCGCTCGAAGGCGTCAGCGTG

sgRNA #2 MYBL2 Top: CACCGGGCCCCGGGCCGCGCTCGA

Bottom: AAACTCGAGCGCGGCCCGGGGCCC

sgRNA luciferase Top: CACCAACGCCTTGATTGACAAGGA

Bottom: AAACTCCTTGTCAATCAAGGCGTT

pCRISPRia-v2:

NTC Top: TTGGGCGATCTAATCGGAACTGGTTTAAGAGC

Bottom: TTAGCTCTTAAACACAGTTCCGATTAGATCGCCCAACAAG

sgRNA #2 MYBL2 Top: TTGGGGGCCCCGGGCCGCGCTCGAGTTTAAGAGC

Bottom: TTAGCTCTTAAACTCGAGCGCGGCCCGGGGCCCCCAACAAG

### RNA interference

For shRNA knockdown sequences, the pRSI9-U6-(sh)-UbiC-TagRFP-2A-Puro backbone (addgene #28289). Sequences were used from ref. [68].

shRNA #1 MYBL2 Top: CTCGAGCTGGTTAAGAAGTAT

Bottom: ATACTTCTTAACCAGCTCGAG

shRNA #2 MYBL2Top: CCAGAAGTACTCCATGGACAA

Bottom: TTGTCCATGGAGTACTTCTGG

### Flow cytometry-based growth competition assay with CRISPRi

For transduction on day −3 500,000 cells were seeded in triplicate on six-well plates in 2 ml of medium and transduced with 1 ml of viral supernatant. For the experiments sgRNAs #1 and sgRNA #2 were used. In addition, cells were transduced with pMCB619 (addgene #171011) as an empty vector (EV) control. Following, the cells were incubated with the viral supernatant for 3 days. On day 0 the cells were sorted using the FACSAria™ Fusion Cell Sorter (BD, Franklin Lakes, NJ, USA) for RFP+/BFP+ cells, respectively. Depending on cell line and transduction efficiency ~100,000–200,000 cells per sample were sorted for qPCR. For the competition assay ~30,000–75,000 cells per transduced sample and the respective amount of the EV-transduced cells needed for all of the sgRNA transductions were sorted. 50% of RFP+ cells and 50% of BFP+ cells were seeded in each well. In the following time, the populations were monitored using flow cytometry at the flow cytometer LSRFortessa™ (BD).

### Flow cytometry-based growth competition assay with shRNAs

For the shRNA competition assay, THP-1 cells were transduced with shRNA #1 and #2 (described in Materials and Methods- RNA interference). The virus was kept on the cells for 3 days, and time point 0 was measured on the same day by flow cytometry, analyzing for RFP+ cells. Three more time points were assessed after initial day 0, and values were normalized to the percentage of RFP+ cells on day 0.

### In vivo shRNA competition assay

Animal experiments were approved according to institutional guidelines and German animal welfare regulations. Mice were bred in-house within the NCT/UCC Dresden animal facility at the University Hospital Dresden in standard individually ventilated cages according to the current hygiene and animal welfare guidelines. For the experiments, NOD/SCID/gamma (NSG) mice (The Jackson Laboratory) were used. Samples from patients with AML were collected from University Hospital Dresden following written informed consent, in compliance with the Declaration of Helsinki. On day 0, PDX cells were lentivirally transduced with scramble shRNA and shRNA #2 MYBL2 using a MOI of around 0.4 to 0.6. The cells were incubated overnight at 37 °C. A day prior to the cell injection, the mice were conditioned with 10 mg/kg busulfan, which was injected intraperitoneally. The next day, the cells were washed and injected in a total volume of 150 µl into the lateral tail vein (five mice per group, groups were randomized with 8–16-week-old mice from both sexes). Blood samples were collected from the facial vein during the course of the experiment for monitoring hCD45+ cell engraftment by flow cytometry. For the staining, the antibodies APC mouse anti-human CD45 (BD Pharmingen ™, 555485) and Brilliant Violet 421™ anti-mouse CD45 (BioLegend, 103133) were used. 40 µl of blood was subjected to 1 × RBC lysis buffer (Invitrogen) for 10 min at RT to ensure lysis of erythrocytes. After centrifugation the cells were resuspended in 100 µl of PBS and stained with 2 µl per antibody for 15 min at RT in the dark. Afterwards, 1 ml of PBS was added and centrifuged. Resuspension was done in 100–200 µl of PBS, and the samples were analyzed by flow cytometry. Once 50% of hCD45+ were reached in the blood, the mice were sacrificed by cervical dislocation, the femur and tibia were removed, and cells were isolated by using a mortar and pestle. The spleen was also removed and crushed by using the piston of a syringe to obtain a cell suspension. The cells from bone marrow, spleen and blood were stained for hCD45+ cells and analyzed for RFP+ cells within the hCD45+ cell population. For the shRNA scramble, three out of five transplanted samples engrafted. Analysis was performed with FlowJo version 10.10.0.

### Library preparation for RNA sequencing and data analysis

As previously described in [69], RNA sequencing (RNA-seq) libraries were prepared using the Stranded mRNA Prep, Ligation Kit (Illumina, San Diego, CA, USA) according to the manufacturer’s instructions. mRNA was purified from 1 µg total RNA using oligo(dT) beads, poly(A) + RNA was fragmented to 150 bp and converted into cDNA, and cDNA fragments were end-repaired, adenylated on the 3’ end, adapter-ligated, and amplified with 12 cycles of PCR. The final libraries were validated using a Qubit 2.0 Fluorometer (Life Technologies) and a Bioanalyzer 2100 system. All barcoded libraries were pooled and sequenced 2 × 75bp paired-end on an Illumina NextSeq550 platform to obtain a minimum of 10 million reads per sample. Quality control assessment of raw reads was performed using FastQC, followed by Trimmomatic trimming to remove low-quality sequences. The trimmed reads were then aligned to the reference genome using STAR version 2.7.9a. To facilitate the mapping process, a genome reference index was constructed using either GRCh37.fa (hg19) or GRCh38.108.gtf (hg38) as the reference. DEG were calculated by comparing the median expression of log2-modified read counts between control and *MYBL2-*knockdown cell lines for all annotated genes. These values were then plotted as log2 fold changes against the logarithmically converted *p*-values obtained from a two-sided unpaired t-test. GSEA was performed using GSEA software (http://www.broadinstitute.org/gsea/) according to the publisher’s instructions [70, 71].

### Senescence assay

For senescence analysis, the Cell Meter Cellular Senescence Activity Assay Kit Red Fluorescence (AAT Bioquest, Inc., Pleasanton, CA, USA) was used following the manufacturer’s protocol. A positive control for senescence was utilized by adding 1 µM Ara-C to cells 48 h prior to each senescence measurement. The cells were incubated with the working solution for 45 min. The cells were then assayed at the flow cytometer LSRFortessa™ (BD). Analysis was performed with FlowJo version 10.10.0.

### Cell cycle and apoptosis analysis

For cell cycle analysis, Hoechst 33342 (Life Technologies) was utilized. Cells were counted, and 500,000 cells were resuspended in 1 ml. Hoechst 33342 was added to the culture medium of the cells at 5 μg/ml and incubated at 37 °C for 60 min. The cells were then washed with PBS, resuspended in 400 µl PBS and measured at the flow cytometer LSRFortessa™ (BD). Annexin V staining was applied to stain apoptotic cells. Here, the APC Annexin V Kit (BioLegend) was used following the manufacturer’s protocol. 150,000 cells were centrifuged and resuspended in 100 µl Annexin V Binding Buffer. 5 µl of APC Annexin V was added, and an unstained control was included that did not receive the dye. Cells were gently vortexed and incubated in the dark at room temperature for 15 min. 300 µl of Annexin V Binding Buffer was added, and the samples were assayed at the flow cytometer LSRFortessa™ (BD). Analysis was performed with FlowJo version 10.10.0.

### Doxycycline-inducible shRNA knockdown

For an inducible shRNA knockdown, we utilized the pLKO-Tet-on-all-in-one system [26] (addgene #21915 Tet-pLKO-puro). We received specific shRNAs targeting *MYBL2* cloned into the vector as described in [68]. Sequences are the same as described for RNA interference. After lentiviral production and infection, cells were selected with puromycin (Sigma-Aldrich) (2 µg/ml). 1 µg/ml puromycin was continuously kept on the cells during cultivation. Knockdown was induced by adding 1 µg/ml doxycycline (dox) to the cells for at least 48 h. To reverse the knockdown, dox was washed off with PBS, and cells were cultured with the usual growth medium again. Senescence analysis was performed as previously described on day 2 and day 12 after initial dox induction. A positive control for senescence was utilized by adding 1 µM Ara-C to cells 48 h prior to each senescence measurement. Additionally, the cells treated with dox were sorted 2 days after induction based on gating on the highest signal for β-galactosidase using the FACSAria™ Fusion Cell Sorter (BD). Around 15,000 cells were sorted per replicate, reseeded into medium and cultured for 3 weeks.

### Analysis of external datasets

Patient data were obtained from the Beat AML cohort from the Oregon Health & Science University (AML, OHSU 2022 Cancer Cell 2022 [28]) and TCGA cohort (AML TCGA [27]). For survival analysis, we used mRNA expression z-scores relative to all samples (log RNA Seq RPKM 671 samples) from Beat AML and mRNA expression (RNA Seq RPKM 179 samples) from TCGA. Patients were stratified into low and high groups based on the median. Raw data were downloaded using the cBioportal online tool (https://www.cbioportal.org/). GSEA was performed using GSEA. software (http://www.broadinstitute.org/gsea/) according to the publisher’s instructions [70, 71].

### Statistical analysis

Two-tailed *t* tests were used to compare differences between groups to assess statistical significance. The results were presented as mean ± standard deviation (SD) from at least three independent experiments. For Kaplan–Meier survival curves, we applied the Log-rank (Mantel–Cox) test. *P* values < 0.05 were considered significant. *P* values are *p < 0.05, **p < 0.01, ***p < 0.001. Data were plotted using GraphPad Prism version 10.2.3 and R version 4.3.2.

## Supplementary information

Supplementary Figures

Supplementary Table 1

Supplementary Table 2

Supplementary Table 3

## Data Availability

The RNA-sequencing data generated in this study have been deposited in the Gene Expression Omnibus (GEO) Repository under accession number GSE305485. All other data supporting the findings of this study are available upon request from the corresponding author.
