# Supplementary material for: Cell cycle regulator MYBL2 is a distinct vulnerability in acute myeloid leukemia
Source: Cell Death Discov. 2025 Oct 20;11:470. doi: 10.1038/s41420-025-02810-4 (PMC12537973; doi:10.1038/s41420-025-02810-4)
Supplement: Supplementary file 1 — Supplementary Figures [file 41420_2025_2810_MOESM1_ESM.docx]

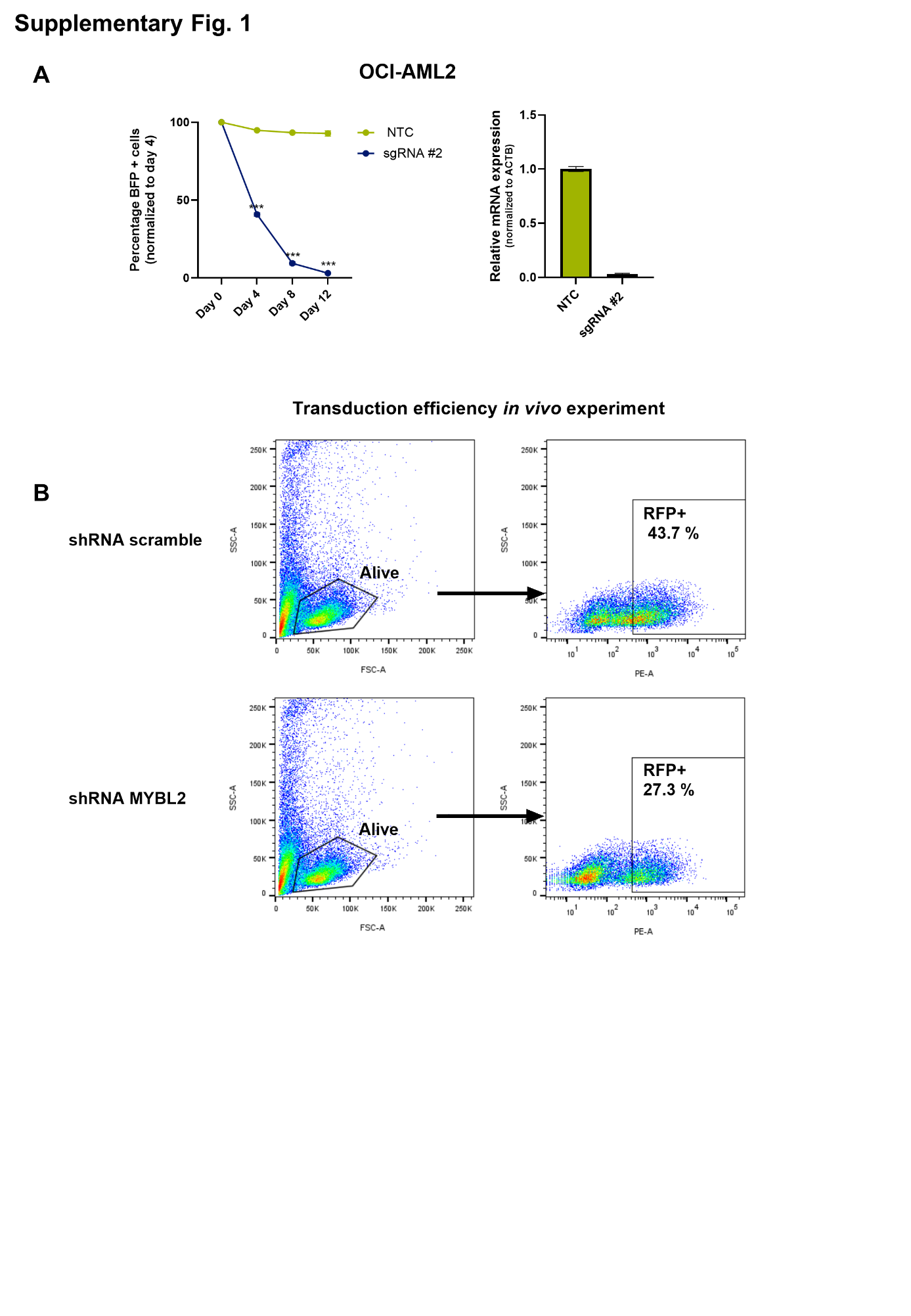


**Supplementary Figure 1- Growth competition assay in OCI-AML2 and transduction efficiency d0 *in vivo* growth competition assay**

(A) CRISPRi growth competition assay of sgRNA transduced OCI-AML2 cells against untransduced cells. Non-targeting control (NTC) was used as a control. Flow cytometry data measured on four different time points showing percentage of BFP expression for sgRNA transduced cells. Indicated p-values were determined by a two-tailed t-test and summarized three independent biological replicates *** p <0.001. mRNA expression of *MYBL2* as measured by qPCR. Expression relative to NTC and normalized to GAPDH. Data show technical triplicates with standard deviation. (B) Flow cytometry plots representing gating strategy and initial transduction efficiency (d0) of cells transplanted for *in vivo* experiment.


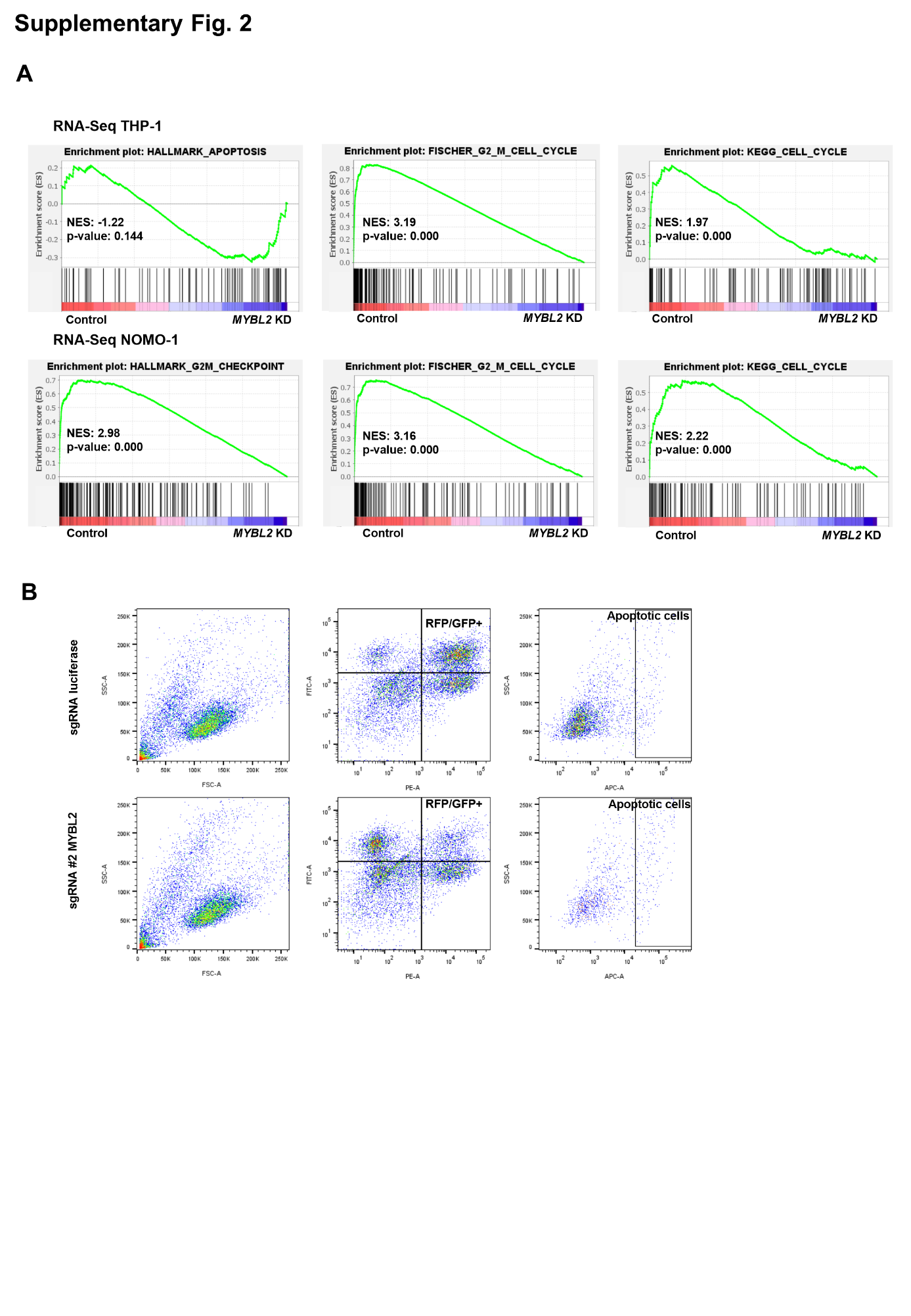


**Supplementary Figure 2- Transcriptomics and apoptosis analysis**

(A) GSEA for RNA sequencing data *MYBL2* knockdown vs control in THP-1 and NOMO-1. (B) Flow cytometry gating strategy for apoptosis analysis representatively for sgRNA luciferase and sgRNA #2 MYBL2 of one replicate.


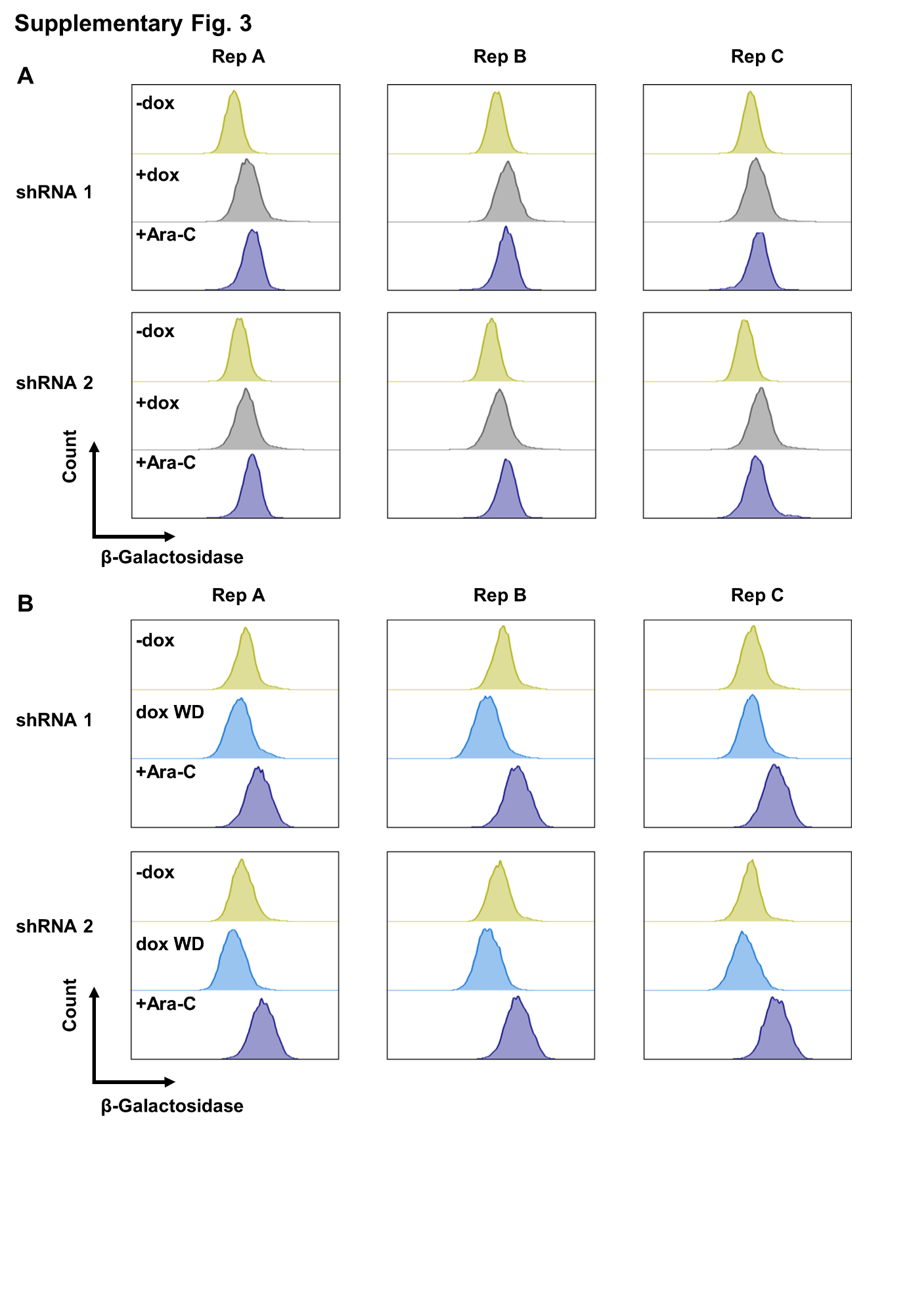


**Supplementary Figure 3- Senescence analysis with knockdown induction and reversal- flow cytometric histograms**

(A) Senescence analysis measuring active β-galactosidase two days after initial dox treatment. Fluorescence intensity was determined by flow cytometry for control (-dox), induction of *MYBL2*-knockdown (+dox) and a positive control, treatment with 1 µM Ara-C for shRNA 1 and shRNA 2 in three independent replicates. Representation as histograms. (B) Senescence analysis measuring active β-galactosidase twelve days after initial dox treatment. Fluorescence intensity was determined by flow cytometry for control (-dox), dox withdrawal (WD) and a positive control, treatment with 1 µM Ara-C for shRNA 1 and shRNA 2 in three independent replicates. Representation as histograms.
